# Decision-making autonomy of women and other factors of anemia among married women in Ethiopia: a multilevel analysis of a countrywide survey

**DOI:** 10.1186/s12889-021-11538-6

**Published:** 2021-08-03

**Authors:** Fentanesh Nibret Tiruneh, Degnet Teferi Asres, Mesfin Wogayehu Tenagashaw, Hirut Assaye

**Affiliations:** Department of Applied Human Nutrition, Faculty of Chemical and Food Engineering, Bahir Dar Institute of Technology, Bahir Dar University, Bahir Dar, Ethiopia

**Keywords:** Anemia, Women’s autonomy, Group-level, Individual-level, Ethiopia

## Abstract

**Background:**

Anemia is one of the world’s public health problem, especially in developing nations. The majority of women of childbearing age (15–49) are affected by anemia. Women’s role in the decision-making process is significant for their health and related issues such as anemia. So far, there is no evidence of women’s decision-making autonomy on anemia. Consequently, this study aimed to robustly examine both individual- and group-level women’s decision-making autonomy and other determinants of anemia among married women in Ethiopia.

**Methods:**

We examined data from an Ethiopian demographic and health survey conducted in 2016. Our analysis included 9220 married women of childbearing age (15–49 years). For bivariate analysis, we applied the chi-squared (X^2^) test. The relationship between individual and group-level women’s decision-making autonomy and anemia was assessed using multilevel binary logistic regression models while adjusting other socio-demographic and economic characteristics.

**Results:**

In this study the magnitude of anemia was 30.5% (95% CI; 29.5–31.4). According to our multilevel analysis, group-level women’s autonomy was found to be negatively related with anemia than individual-level women’s autonomy (AOR = 0.53, 95% CI = 0.41–0.69). In addition, the indicator of women’s wealth index at group level was a protective factor (AOR = 0.68, 95% CI =0.51–0.90) to develop anemia. Among individual-level indicators women’s age (AOR = 0.73, 95% CI = 0.60–0.89), use of contraceptive (AOR = 0.66, 95% CI = 0.55–0.81), BMI (AOR = 0.71, 95% CI = 0.59–0.86) and employment status (AOR = 0.88, 95% CI = 0.79–0.98) were negatively related with anemia. While women who follow Muslim religion (AOR = 1.62, 95% CI = 1.32–1.97,), women who had five and above number of children (AOR = 93, 95% CI = 1.53–2.46), and who were pregnant (AOR = 1.21, 95% CI = 1.04–1.40) were positively associated with anemia.

Our final model showed that around 27% of the variability of having anemia was because of group-level differences (ICC = 0.27, *P* < 0.001). In addition, both individual and group-level factors account for 56.4% of the variance in the in the severity of anemia across communities (PCV = 56.4%).

**Conclusions:**

Our study showed that empowering women within households is not only an important mechanism to reduce anemia among married women but also serves as a way to improve the lives of other women within the society**.**

**Supplementary Information:**

The online version contains supplementary material available at 10.1186/s12889-021-11538-6.

## Background

Anemia occurs when the number of red blood cells of the body is insufficient to meet the body’s physiological needs. This is achieved by a hemoglobin concentration of less than 110 g/L for pregnant women and 120 g/L for non-pregnant women [1]. Globally about 29.4% of women of childbearing age and 38.2% of pregnant women are anemic. The problem is most common in developing nations which accounts for more than 89% [2, 3].

In Africa, more than 32.8% of women of childbearing age are anemic and in some African countries, the prevalence is far above 50% [3, 4]. Although anemia control is a priority public health agenda in Ethiopia, 24% of women of childbearing age group are anemic [5].

Numerous studies have envisaged lots of potential determinants of anemia among women particularly, repeated to births, residence, pregnancy status, nutritional status, age, and breastfeeding status [6–9]. However, there is the literature gap in the relationship between women’s decision-making autonomy and the magnitude of anemia among them.

Autonomy is a multifaceted concept that encompasses certain powers to manage the personal environment by controlling resources and information, including freedom of movement, making decisions about one’s personal concerns, or by close family members [10, 11]. Women’s autonomy is important in their own right and is crucially linked to women’s health and health-related issue [12]. Evidence suggests that increasing the access of economic resources alone is insufficient but requires increased women’s decision-making and control of household resources is also highly required. In order to practice good health-seeking behaviors, women’s ability to participate in decision-making over certain important matters (such as major household purchases or personal health care) is essential [13, 14].

Policymakers and researchers have called for an emphasis on improving women’s decision-making power to improve women’s health and health-seeking behaviors in developing countries [15]. Studies in South Asia and sub-Saharan African countries indicated that women’s decision-making autonomy has been linked to many positive outcomes such as reductions in infant mortality, better child and women nutritional outcomes, and increased use of health care services [16, 17].

Some studies indicated that the decision-making capabilities of women in a society might have also an effect on their health and health-related behavior, more than the effect of individual women’s autonomy for two major reasons [18]. The first reason is that the presence of high numbers of a greater proportion of empowered women in a community could contribute to the information distribution related to better health outcomes to those with lower autonomous levels through formal and informal social networks [19]. The other reason is that communities with a higher proportion of autonomous women might be economically advantaged because more women are employed (working outside of the home). This will empower them to make better investments in the health sector creating an opportunity for less autonomous women more access to health information and services for themselves and their families [20].

In Ethiopia, although several pocket studies have examined the determinants of anemia, they largely focused only on individual-level characteristics and did not examine community determinants. To our knowledge, none have accounted for both individual and group-level women’s autonomy related to anemia. Moreover, information that used a national representative sample and factors at the group-level that may have an effect on anemia among married women is typically overlooked.

Therefore, this study aimed to more robustly examine individual- and group-level women’s decision-making autonomy and other factors of anemia amongst married women in Ethiopia. Being based on data from a nationally representative sample, this study will give imminent information for concerned stakeholders and policymakers to understand the determinants of anemia in order to design and implement feasible interventions both at individual and group levels. We hypothesized that women with a high level of decision-making autonomy at both the individual and group-levels are less likely to develop anemia than were their counterparts.

## Methods

### Study sample

Data obtained from the 2016 Ethiopia Demographic and Health Surveys (EDHS) was used for this study. The 2016 EDHS is a nationally representative database compiled by the National Central Statistical Agency (CSA). It is intended to generate representative assessments for the majority of national demographic and health indicators [5].

**Fig. 1:**
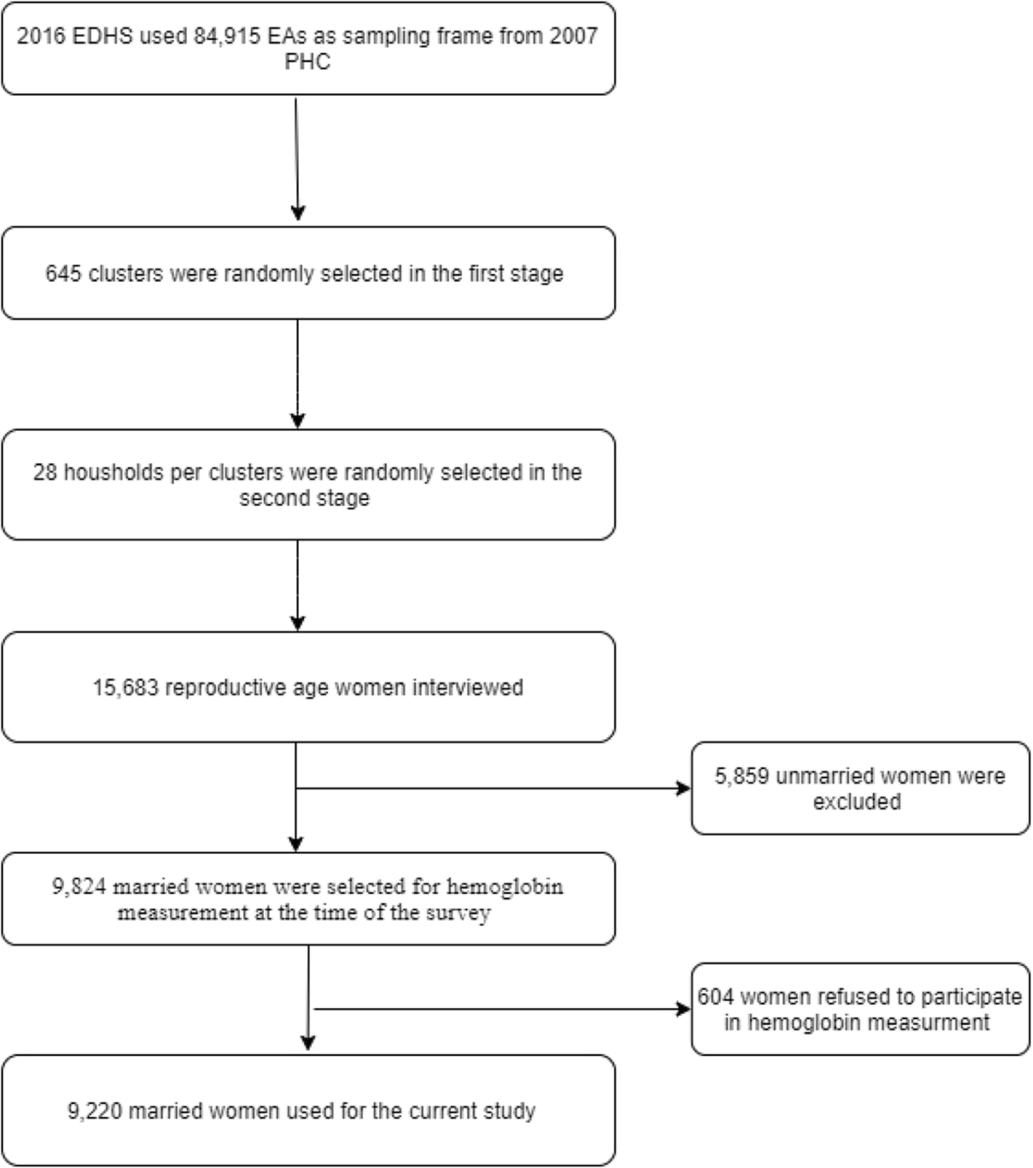
Sampling procedure; from 2016 Ethiopian Demographic and Health Survey (EDHS)

Households were chosen by using stratified two-stage cluster sampling. In the first phase, a total of 645 clusters/enumeration areas (EAs) (202 in urban areas and 443 in rural areas) from a list of 84,915 EAs created for the 2007 Ethiopian Population and Housing Census (PHC) were randomly selected. In the second phase, 28 households were randomly selected for each cluster. In the 2016 EDHS, a cluster was defined as a census enumeration area covering 181 households on average. Of the 15,683 women interviewed, 5859 women were excluded because they were single. Among 9824 married women who selected for hemoglobin measurement, 604 women refused to participate in hemoglobin measurement. As a result, this study concentrated on 9220 married women of childbearing age (15–49 years) in 645 clusters who were tested for their hemoglobin level (Fig. 1).

Furthermore, face-to-face structured interviews were conducted. Before the survey, a pretest was conducted to ensure that the questions were clear for the study participants.

#### Measures

##### Outcome variable

Anemia among married women is the outcome variable, measured as a categorical variable through predefined cut-off points for mild (110–119 g/dl for non-pregnant women, 100–109 g/dl for pregnant women), moderate (80–109 for non-pregnant women, 70-99 g/dl for pregnant women), and severe anemia (lower than 80 g/dl for non-pregnant women, lower than 70 for pregnant women) as recommended by WHO [21]. However, due to the small number of cases in our dataset that fall into the categories of severe and mild anemia, we recoded anemia levels as “0” for non-anemic and “1” for anemic [22].

##### Individual-level independent variables

We selected the individual-level variables from the existing literatures (10, 12, 16, and 19).

Five key questions were used to assess women’s decision-making autonomy. (a) Who makes health-care decisions for women? (Personal decision-making autonomy), (b) who makes decision on large household purchases (economic decision-making autonomy), (c) who decides on visits to friends or family (mobility decision-making autonomy), (d) who makes the decision about what to do with the husband’s earnings and (e) who decides to use contraception. Following these questions, women, who made all of the above decisions, either alone or with her husband, were classified as having high decision-making autonomy, whereas the other women were classified as having low decision-making autonomy [23].

Other individual-level variables included in our study were respondents’ age (15–24,

25–34, and 35–49 years); age of first marriage (< 16, 16–20 and > 20), religion (Orthodox, protestant Muslim and others), level of education (no education, primary, secondary and higher), employment status (no/yes), education level of husband (no education, primary, secondary and higher), place of residence (urban/rural), number of children ever born (0,1-2,3–4 and + 5), current pregnancy (no/yes), currently breastfeeding (no/yes), amenorrheic (no/yes) at the time of data collection, women’s BMI (underweight, normal weight and overweight), history of terminated pregnancy (no/yes), health insurance coverage (no/yes), whether women chewed chat (no/yes) and health service utilization within 1 year (no/yes). The wealth index was an integrated measure of household properties such as televisions, bicycles, building materials, access to water, sanitation facilities and other wealth-related aspects. Factor scores of household assets were developed using principal component analysis. The lowest 45.5% of the scores were classified as poor, while the remaining scores were classified as non-poor [24].

##### Community-level independent variables

A community (cluster) is defined as a census enumeration area covering 181 households on average [5]. In this study, we included five group-level variables. One variable indicates an area of residence, (place of residence). Four group-level variables were obtained by combining individual responses for each item to the group level. These were the percentage of women with a high level of decision-making autonomy, attained higher education, had a non-poor wealth index and had ever chewed chat in the community. On the basis of tertiles, each combined variable was classified as low, medium, or high. The selected community-level variables represented the norms and social contexts of the community regarding women’s decision-making autonomy, the availability of health related resources, and the socioeconomic status of the community [25].

## Statistical examinations

All statistical examinations were done using SAS 9.4. Distribution of the respondent’s individual and group-level characteristics with anemia was assessed using Chi-square tests. Those explanatory variables that showed significance (*p* < 0.25) in the x^2^-square analysis were included for multivariate analysis. Multilevel binary logistic regression models were constructed by using the “GLIMMIX” command in SAS to simultaneously analyze the relationships of the individual- and group-level factors with anemia.

### Modeling approaches

Three modeling methods were used: fixed effects, random effects, and goodness of fit.

#### Fixed effect

The multilevel binary logistic regression model’s fixed effects were reported as adjusted odds ratios (AORs) with 95% confidence intervals (CI).

#### Random effect

Community variance in each model was assessed by using the intraclass correlation coefficient (ICC) and proportional change in variance (PCV).

$$ \mathrm{ICC}=\frac{\mathit{\operatorname{var}}(b)}{\mathit{\operatorname{var}}(b)+\mathit{\operatorname{var}}(w)}, $$

Where Var(b) is the group level variance. Var (w) is the standard logistic distribution, which is the assumed individual variance component, which is $\frac{\pi^2}{3}$ ≈3.29 [26].

$$ \mathrm{PCV}=\frac{Est0- Estimate\ for\ model\ \left(\hat{\mkern6mu} \right)}{\mathrm{Est}0}\ast 100. $$

Where Est0 is an estimate from null model and (^) is an Estimate from model 1, 2, and 3.

There were four models produced. For entering variables into the multivariate models, we used a hierarchical approach. The initial model (null model) comprised only the outcome variable to examine the variation in anemia across communities/groups. The second model (model with only individual-level variables), the third model (model with only group-level variables) and the final multilevel model include both individual level and group level variables that are fitted with anemia at the same time.

#### Goodness of fit

Each model’s goodness-of-fit was evaluated by using the Bayesian Information Criteria (BIC), a lower value indicates that the model is better fit. To assess multicollinearity, the variance inflation factor (VIF) and tolerance were used. None of the variables showed signs of multicollinearity (all VIF < 10 and all tolerance > 0.1) (See supplementary Table 1).

## Ethical considerations

The survey protocol, which included biomarker collection, was reviewed and approved by the Ministry of Innovation and Technology, the Federal Democratic Republic of Ethiopia, and the ICF International Institutional Review Board. At the start of data collection, participants provided informed consent, and the authors sought permission from the DHS program to use the data: https://dhsprogram.com/data/dataset/Ethiopia_Standard-DHS_2016.cfm?flag=0

## Results

The magnitude of anemia among married women in our study was 30.5% (95% CI: 29.5–34.4). About 39% of respondents had high decision-making autonomy at household level (Table 1). Table 1 also shows information about the sampled women’s socioeconomic and demographic characteristics. The majorities of women were from rural areas, had no formal education, and were unemployed. Regarding household wealth status, 45.5 and 54.5% of study respondents were from poor and non-poor households,’ respectively. Approximately 65% of the married women had normal nutritional status (BMI = 18.5–24.9). Furthermore, only 4.1% of participants had health insurance.

**Table 1:** Characteristics of sample married women in the Ethiopia 2016 Demographic and Health Survey (EDHS) sample

	Frequency	%
***outcome variable (Anemia)***		
None anemic	6412	69.5
Anemic	2808	30.5
**Individual-level Characteristics(*n*** **= 9220)**		
*Women’s autonomy*		
Low	7196	61.0
High	3597	39.0
Socio demographic and economic characteristics		
Women’s Age		
15–24	2294	24.9
25–34	3852	41.9
35–49	3074	33.3
Women’s age at first Marriage		
< 16	3519	38.2
16–20	4194	45.5
> 20	1507	16.3
Religion		
Orthodox	3337	36.2
Protestant	1706	18.5
Muslim	3998	43.4
Others	179	1.9
Women’s education level		
No Education	5387	58.4
Primary	2553	27.7
Secondary	805	8.7
Higher	475	5.1
Education level of husband or partner		
No education	4192	45.5
Primary	2905	31.5
Secondary	1126	12.2
Higher	911	9.9
Women’s employment		
No	6267	67.9
Yes	2953	23.1
Currently pregnant		
No	8199	88.3
Yes	1021	11.1
Currently breastfeeding		
No	5379	58.3
Yes	3841	41.7
Currently amenorrheic		
No	6753	73.2
Yes	2467	26.8
Number of children ever born		
0	889	9.6
1–2	2749	29.8
3–4	2205	23.9
5+	337	36.6
Current use of contraceptive		
No	6402	69.4
Yes	2818	30.6
Women ever chewed Chat		
No	8174	88.7
Yes	1046	11.3
Wealth Index		
Poor	4198	45.5
Non-Poor	5022	54.5
History of terminated pregnancy		
No	8233	89.3
Yes	987	10.7
Women’s BMI		
< 18.5(underweight)	2078	22.5
18.5–24.9(normal weight)	6010	65.2
≥ 25(overweight)	1132	12.3
*Health care access*		
Covered by health insurance		
No	8841	95.9
Yes	379	4.1
Visiting health facility in the last 12 months		
No	4558	49.4
Yes	4662	50.6
Community-level characteristics		
Place of residence		
Urban	2229	24.2
Rural	6991	75.8
% of women with high decision-making autonomy		
Low	226	35.0
Medium	152	23.5
High	267	41.4
% of women with non-poor wealth index		
Low	196	30.4
Medium	184	28.5
High	265	41.1
% women with higher education		
Low	228	35.3
Medium	255	39.5
High	162	25.1
% women who chewed chat		
Low	315	48.8
Medium	173	26.8
High	157	24.3

The findingss of the bivariate analysis of both individual- and group-level factors and anemia prevalence were presented in (Table 2). Women’s decision making autonomy at household, age, age at first marriage, religion, educational level, educational level of husband, employment status, pregnancy and breastfeeding status, number of children ever born, contraceptive use, wealth index, nutritional status, insurance coverage and whether women visit the health facility in the last 12 months were individual level factors that showed statistically significance difference with anemia. Place of residence, community-level women’s decision-making power, education, wealth index, were also showed significantly associated with anemia among community-level factors.

**Table 2:** Bivariate analyses of individual and group-level factors with Anemia among married women in Ethiopia

	Anemic		*P*-value
	No%	Yes%	
**Individual-level Characteristics(*n*** **= 9220)**			
*Women’s autonomy*			
Women’s decision-making power at home			< 0.001
Low	50.8	24.7*	
High	20.0	4.5	
Socio demographic and economic characteristics			
Women’s Age			
15–24	16.9	6.8*	0.048
25–34	29.5	12.5	
35–49	24.4	9.9	
Women’s age at first Marriage			< 0.001
< 16	25.0	10.8*	
16–20	31.3	13.4	
> 20	14.5	5.0	
Religion			< 0.001
Orthodox	32.2	7.5*	
Protestant	12.9	5.4	
Muslim	24.4	15.9	
Others	1.35	0.4	
Women’s education level			< 0.001
No Education	32.2	16.8*	
Primary	22.8	7.9	
Secondary	9.8	3.0	
Higher	6.0	1.4	
Education level of husband or partner			0.003
No education	27.64	15.2*	
Primary	23.4	8.6	
Secondary	10.0	3.6	
Higher	8.5	2.4	
Women’s employment			< 0.001
No	44.0	20.7*	
Yes	26.9	8.5	
Currently pregnant			< 0.001
No	65.15	25.7*	
Yes	5.7	3.4	
Currently breastfeeding			< 0.001
No	47.3	18.5*	
Yes	23.5	10.7	
Currently amenorrheic			< 0.001
No	56.5	22.0	
Yes	14.3	7.2	
Number of children ever born			< 0.001
0	8.5	2.2*	
1–2	24.5	8.3	
3–4	15.9	6.9	
5+	22.0	11.8	
Current use of contraceptive			< 0.001
No	43.55	22.8*	
Yes	27.3	6.4	
Women ever chewed Chat			0.064
No	63.3	26.0	
Yes	7.5	3.2	
Wealth Index			< 0.001
Poor	23.6	14.8*	
Non-Poor	47.2	14.4	
History of terminated pregnancy			0.039
No	63.9	26.5	
Yes	6.9	2.7	
Women’s BMI			< 0.001
< 18.5(underweight)	13.01	7.4*	
18.5–24.9(normal weight)	46.5	18.0	
≥ 25(overweight)	11.3	3.8	
*Health care access*			
Covered by health insurance			< 0.001
No	67.2	28.4*	
Yes	3.6	0.8	
Visiting health facility in the last 12			< 0.001
No	35.15	16.5*	
Yes	35.7	12.7	
**Community-level characteristics**			
Place of residence			
Urban	26.6	7.5*	
Rural	44.2	21.7	
% of women with high decision-making autonomy			
Low	17.5	14.3*	< 0.001
Medium	18.2	6.2	
High	35.1	8.7	
% women with higher education			< 0.001
Low	22.0	12.5*	
Medium	30.3	9.9	
High	18.5	6.7	
% of women with non-poor wealth index			< 0.001
Low	15.2	11.5*	
Medium	20.9	7.9	
High	34.8	9.8	
% women who chewed chat			0.347
Low	47.6	19.9	
High	23.2	9.3	

* P < 0.05

Table 3 displays the findings from the multilevel analyses. The findings of the empty model (model1), which only included the outcome variable, confirmed a significant variance in the magnitude of anemia among women across communities. Group level factors accounted for 46% of the total variance in anemia prevalence (ICC = 0.46, *p* < 0.001).

**Table 3:** Multilevel analyses of factors associated with anemia, among married women in Ethiopia

Variables	Model l	Model 2 AOR(95%CI)	Model 3 AOR(95%CI)	Model 4 AOR(95%CI)
**Individual -level characteristics(*n*** **= 9220)**				
*Women’s autonomy*				
Women’s decision-making power at home				
Low		1.00		1.00
High		0.95 (0.72–1.17)		0.97 (0.79–1.21)
Socio demographic and economic characteristics				
Women’s Age				
15–24		1.00		1.00
25–34		0.867 (0.74–1.01)		0.88 (0.76–1.03)
35–49		0.71 (0.58–0.86)*		0.73 (0.60–0.89)*
Women’s age at first Marriage				
< 16		1.00		1.00
16–20		1.06 (0.95–1.18)		1.05 (0.95–1.17)
> 20		1.15 (0.98–136)		1.14 (0.97–1.34)
Religion				
Orthodox		1.00		1.00
Protestant		1.44 (1.17–1.77)*		1.37 (1.11–1.68)*
Muslim		1.92 (1.60–2.31)*		1.62 (1.32–1.97)*
Others		1.12 (0.74–1.69)		1.02 (0.67–1.53)
Women’s education level				
No Education		1.00		1.00
Primary		0.89 (0.79–1.01)		0.89 (0.45–1.28)
Secondary		1.03 (0.84–1.27)		1.04 (0.84–1.28)
Higher		0.87 (0.65–1.16)		0.87 (0.65.1.16)
Education level of husband or partner				
No education		1.00		1.00
Primary		0.95 (0.83–1.06)		0.95 (0.85–1.08)
Secondary		1.10 (0.92–1.32)		1.10 (0.92–1.32)
Higher		0.99 (0.80–1.24)		0.99 (0.79–1.24)
Women’s employment				
No		1.00		1.00
Yes		0.88 (0.79–0.89)*		0.88 (0.79–0.98)*
Currently pregnant				
No		1.00		1.00
Yes		1.21 (1.04–1.40)*		1.21 (1.04–1.40)*
Currently breastfeeding				
No		1.00		1.00
Yes		1.06 (0.94–1.20)		1.06 (0.94–1.20)
Currently amenorrheic				
No		1.00		1.00
Yes		1.01 (0.89–1.14)		1.01 (0.88–1.14)
Number of children ever born				
0		1.00		1.00
1–2		0.46 (1.21–1.77)*		1.45 (1.20–1.75)*
3–4		1.65 (1.33–2.06)*		1.63 (1.31–2.03)*
5+		2.01 (1.57–2.53)*		1.93 (1.53–2.46)*
Current use of contraceptive				
No		1.00		1.00
Yes		0.64 (0.53–0.78)*		0.66 (0.55–0.81)*
Women ever chewed Chat				
No		1.00		1.00
Yes		0.97 (0.82–1.15)		0.98 (0.83–1.17)
Wealth Index				
Poor		1.00		1.00
Non-Poor		0.82 (0.72–0.94)*		0.91 (0.79–1.04)
History of terminated pregnancy				
No		1.00		1.00
Yes		0.93 (0.80–1.08)		0.93 (0.80–1.08)
Women’s BMI				
< 18.5		1.00		1.00
18.5–24.9		0.88 (0.79–0.99)*		0.89 (0.80–1.08)
≥ 25		0.70 (0.58–0.85)*		0.71 (0.59–0.86)*
*Health care access*				
Covered by health insurance				
No		1.00		1.00
Yes		1.08 (0.82–1.42)		1.12 (0.85–1.48)
Visiting health facility in the last 12				
No		1.00		1.00
Yes		0.95 (0.6–1.04)		0.96 (0.87–1.06)
**Community-level characteristics**				
Place of residence				
Urban			1.00	1.00
Rural			1.47 (0.98–2.19)	1.10 (0.82–1.46)
% of women with high decision-making autonomy				
Low			1.00	1.00
Medium			0.36 (0.25–0.51)*	0.54 (0.40–0.73)*
High			0.26 (0.18–0.38)*	0.50 (0.47–0.69)*
% women with higher education				
Low			1.00	1.00
Medium			0.72 (0.53–0.97)*	0.69 (0.54–1.09)
High			0.93 (0.65–1.32)	0.89 (0.66–1.20)
% of women with non-poor wealth index				
Low			1.00	1.00
Medium			0.70 (0.49–1.01)	0.76 (0.56–1.03)
High			0.66 (0.41–0.85)*	0.78 (0.52–0.97)*
Measures of variation (random effect)				
ICC	0.46*	0.29*	0.39*	0.27*
PCV (%)	Ref.	50.7	23.2	56.4
Model fit statistics				
BIC	14,845.3	13,538.1	14,722.2	13,484.7

*AOR* Adjusted odds ratio, *CI* confidence interval, *ICC* intraclass correlation coefficient, *BIC* Bayesian information criterion, *: Significant at *P*-value < 0.05. Note: Boldface indicates the statistical significance values

Model 2 which included outcome and only individual-level variable, showed that women’s age, region, place of residence, employment status, pregnancy status, use of contraception, household wealth index and BMI were significantly related to anemia; the ICC revealed that 29% of the difference in the prevalence of anemia among married women was because of the variation at the group level factors (ICC = 0.29, *p* < 0.001). According to PCV, individual level factors account for 50.7% variation in the odds of having anemia across communities.

The analysis of the outcome and only group-level variables presented in model 3 indicated that group-level women’s decision-making autonomy and wealth index were significantly associated with anemia. The result confirmed that 39% of the variation of anemia was due to the community differences (ICC = 0.39, *p <* 0.001). Community- level factors account for 23.2% of the variation in the odds of having anemia across communities (PCV = 23.2%).

In the final model (model 4), including the outcome variable and both the individual and group-level characteristics at the same time, showed that the risk of anemia was 27% (AOR = 0.73; 95% CI = 0.60–0.89) lower among older women than younger women. Women who follow Muslim religions had 1.62 times higher risk of having anemia (AOR = 1.62, 95% CI = 1.32–1.97) than women who follow orthodox religion. When compared to unemployed women, employed women had a 12% lower risk of anemia (AOR = 0.88, 95% CI = 0.79–0.98). Women who became pregnant during the data collection period were 1.21 times (AOR = 1.21, 95% CI = 1.04–1.40) more likely to have anemia than their counterparts. Women who had more than five children had 1.93 (AOR = 1.93, 95% CI 1.53–2.46) times the odds of having anemia as compared to women who did not have a child. Women who used contraceptive methods at the time of data collection had a 34% (AOR = 0.66; 95% CI = 0.55–0.81) lower risk of anemia. Regarding nutritional status of women, Anemia was found to be 0.71 times less likely in overweight women than in underweight women (AOR = 0.71, 95% CI = 0.59–0.86).

Among group-level factors, women’s decision-making power and wealth index were statistically related with anemia. The chance of having anemia were 50%(AOR = 0.50, 95% CI = 0.47–0.69) and 22% (AOR = 0.78, 95% CI = 0.52–0.98) lower among women in a group with agreater percentage of women who had high decision-making power and non-poor wealth index, respectively than their counterparts. Even after the inclusion of both individual and group-level indicators, the difference of having anemia across communities continued significant; as demonstrated by the estimated ICC, 27% of the variation of having anemia was due to community differences (ICC = 0.27, *P* < 0.001). Furthermore, according to the PCV, 56.4% of the difference in the likelihood of having anemia across communities is described by both individual and community-level determinants (Table 3).

## Discussion

Our findings revealed that the magnitude of anemia among married women in Ethiopia was relatively high 30.5% (95% CI = 29.5–31.4) compared to those reported in the nationwide study among lactating and reproductive age of women [5, 22]. This indicates that married women are at a greater risk of anemia. The majority of respondents (61%) in our sample had low decision-making autonomy at home. Therefore, the greater magnitude of anemia in our sample could be due to the low decision-making capacity of women to access resources and health information. Gender power dynamics influence how and who makes health-related decisions [27].) In families and communities, this power dynamics can affect health-seeking processes such as healthy eating patterns and health outcomes. Globally and especially in sub-Saharan African countries such as Ethiopia, men frequently make decisions regarding their wives’ health [28, 29].. Poor women’s welfare is a barrier to reaching the eventual aim of universal health. This suggests that substantial work is required to decrease the magnitude of anemia among married females, as it has a negative impact on the well-being of mothers, children, and the rest of the family.

This study has determined that in comparing the impact of individual-level women’s decision-making with anemia, the effect of group level decision making power was considered significant. Women living in a society with a high percentage of women with high decision-making status were less likely to develop anemia even after we controlled their decision-making status and other factors at the individual and group level. This may be because social interactions in Ethiopia are often intertwined and therefore, the effect of group group-level autonomy on individual behavior may exceed the individual’s autonomy [30]. The group level may indicate that even though Ethiopian women have low decision-making power, living in a strong decision-making society for women can lead to increased use of shared information and resources and ultimately, help improve the quality of life for women and their family members as well [18]. Female decision-making power at the group level had a beneficial effect on lowering the risk of anemia, implying that gender disparity, as measured by low decision-making power, may influence the development of anemia among married women.

In addition to group-level women’s autonomy, our analyses revealed that the group-level wealth index had a greater impact than the effects of the individual-level wealth index on anemia. A high group-level wealth index was related to reduce odds of having anemia. Prior studies also demonstrated the positive relationship between community economic status and women’s health; economically poorer communities have deficient health information and services [31, 32]. Therefore, interventions aimed at empowering women economically at the group level should be considered to reduce the prevalence of anemia and other health-related problems.

Unlike the findings of other studies [6, 9, 33, 34], our study found that older women had a lower risk to develop anemia than younger ones. The possible reason for this finding is that during adolescence, pregnancy and lactation, younger women have increased iron requirements and to compensate for menstruation-related blood loss [35]. The high percentage of early married women in our sample may provide another possible explanation. Our study found that more than 83% of women were married before the age of 20 years and according to our bivariate analysis, the prevalence of anemia among them was also significantly higher (10.8% vs. 5.0%). %). In Ethiopia, child marriage is common, and decisions about having children and using birth control are often made by husbands [36, 37]. Furthermore, the customs of Ethiopian society strongly oppose the postponement of the first child after marriage, leading to a higher rate of adolescent birth. Therefore, the greater magnitude of anemia among younger females might be because of adolescent pregnancy and childbirth. This implies interventions such as educating the parents and community members who eventually bear the majority of determinations about girls marriage; empowering girls with information (such as exposure to media) about the consequences of early marriage and pregnancy, improving the accessibility and standard of formal education for girls must be prioritized in order to preclude girls marriage and having children. Furthermore, all females of childbearing age need to take iron and folic acid supplements to avoid anemia [38].

In this study, we found that when compared to orthodox followers, Muslim and Protestant women were 37 and 62% more likely to develop anemia, respectively. The possible reason for this result may be due to different dietary patterns among different religious followers. In Ethiopian Orthodox Church, more than 200 days including every Wednesday and Friday in a year are dedicated to religious fasting, which includes abstaining from all types of animal-based foods [39]. Good plant sources of iron including legumes (beans, peas, and lentils), pumpkin, flaxseeds, and vegetables such as spinach, beets and chard are common foods in the fasting dishes. Moreover, Shiro wot, basically made from ground beans/chickpeas, and is also particularly popular as a fasting food among orthodox followers in Ethiopia and relatively inexpensive. Thus, women may get enough non-heme iron from plant-based foods. A longitudinal study conducted in Greek also reported that orthodox fasters had significantly higher iron intake at the end of the fasting period compared to non-fasters [40]. Another potential explanation for the greater magnitude of anemia amongst Muslim females, perhaps because of large family sizes [41–43]. An additional reason may be the frequent consumption of milk and milk product (attribute to iron absorption inhibitor) among Muslim women because most of them are living in the lowlands of the eastern part of Ethiopia (Afar and Somali regions) mostly live on pastoralism. Consumption of iron-rich cereals such as Teff (*Eragrostis tef*), which is common in the highlands of Ethiopia is also rare in these regions of the country [44]. However, further studies regarding the relationship between religion and anemia status in Ethiopia are worth doing.

Our findings supported the findings of other studies by demonstrating that female employment was negatively associated with anemia [22, 45, 46]. The lower prevalence of anemia among working women could indicate financial advantage as women who were employed can earn relatively a higher wage and purchase a wider range of foods including iron-rich ones [47]. Another reason could be that married women who are employed have better decision-making autonomy to receive health-related information and the ability to practice a healthy dietary pattern [22].

This study showed that pregnancy augmented the likelihood of anemia occurrence among married females. During pregnancy, the volume of bloodlines increases, consequently iron and vitamins are needed to make more red blood cells [48].. Greater fluid is produced to help fetal growth and development, however this is only feasible if the body can bulid the desired number of red blood cells; these physiological alterations rise the threat of anemia, particularly if the woman’s nutritional desires are not met [49]. Pregnant ladies who are anemic have poor pregnancy consequences such as low birth weight and stillbirths, and can even result in the mother’s death [50]. This suggests emphasizing proper nutrition and iron supplement for women, especially during pregnancy.

This study found that, women who have ever given birth have a higher risk of developing anemia than women who have never had a child. Moreover, the prevalence of anemia increased with an increased number of children. This might be related with a reduction of maternal iron reserves at every pregnancy and blood loss at each delivery. Another possible reason may be due to the fact that increasing family size could be linked with food insecurity. This finding is consistent with other previous study findings [51, 52].

Contraceptive use was found to be inversely related to anemia in our study. A possible explanation is that contraception use can have a significant impact on achieving good nutrition outcomes by assisting women in delaying pregnancy, spacing births, and reducing a woman’s fertility. Well-spaced births allow women’s bodies to recover and replenish essential nutrients such as iron, resulting in better nutritional outcomes [53]. The inverse relationship between contraceptive use and anemia has important implications for educating healthcare providers and women about the additional nutritional benefits of contraception use [54].

In this study, underweight women were more likely to suffer from anemia compared to overweight women. Studies conducted in Nepal, South Asia, Jordan, Japan and Rwanda [55–59], also reported similar findings. Women who are malnourished are a greater risk to be insufficient in crucial nutrients including iron, which may be linked with an expanded the threat of anemia [60]. Based on our findings, we suggest that anemia prevention interventions in Ethiopia must focus on encouraging secure nutritional exercises to keep a healthy body mass index and endorse iron supplements to confirm ideal nourishment among women, because overweight/obesity is an emerging problem in developing countries, including Ethiopia.

### Limitations

This research has some limitations. First, due to the nature of a cross-sectional study design, we were unable to establish cause-and-effect relationships among explanatory and outcome variables. Second, due to the fact this study is based on secondary records analysis, we are not able to account for all confounding factors. Third, the use of administratively defined boundaries has possible to introduce misclassification for improper administrative classifications. Fourth as most previous empirical studies, our study not completely integrated theory into our conceptualizations and selection of factors. As a result, some measurements might be inaccurate or biased. In spite of these limitations, this study employed a large sample of married women in Ethiopia; consequently our findings may be representative of Ethiopian married women.

## Conclusion

This study underscores the importance of individual and group-level factors in assessing women’s decision-making autonomy and anemia prevalence. Group-level women’s decision-making autonomy and wealth index at the group-level were important factors associated with the prevalence of anemia among married women in Ethiopia. Women’s, age, religion, number of children ever born, use of contraceptive, nutritional status, employment and pregnancy status also important factors linked to anemia. The negative relationship between group-level women’s decision-making autonomy with anemia may suggest a requirement for implementing programs and policies to increase women’s decision-making autonomy in Ethiopia. The government can focus on community awareness regarding women’s rights related to their health. Interventions to increase the economic status of women at the community level should be also implemented.

## Supplementary Information

**Additional file 1: Table 1**. Multicollinearity examination.

## Data Availability

The datasets used in this study can be found at: http://dhsprogram.com/data/Access-Instructions.cfm

## Abbreviations

AOR: Adjusted Odd Ratio
BIC: Bayesian Information Criterion
BMI: Body mass index
CI: Confidence Interval
CSA: Central Statistical Agency
EAs: Enumeration Areas
EDHS: Ethiopia Demographic Health Survey
ICC: Intraclass Correlation Coefficient
SAS: Statistical Analysis Software
WHO: World Health Organization
