# Supplementary material for: Decision-making autonomy of women and other factors of anemia among married women in Ethiopia: a multilevel analysis of a countrywide survey
Source: BMC Public Health. 2021 Aug 3;21:1497. doi: 10.1186/s12889-021-11538-6 (PMC8336366; doi:10.1186/s12889-021-11538-6)
Supplement: Supplementary file 1 — Additional file 1: Table 1. Multicollinearity examination. [file 12889_2021_11538_MOESM1_ESM.docx]

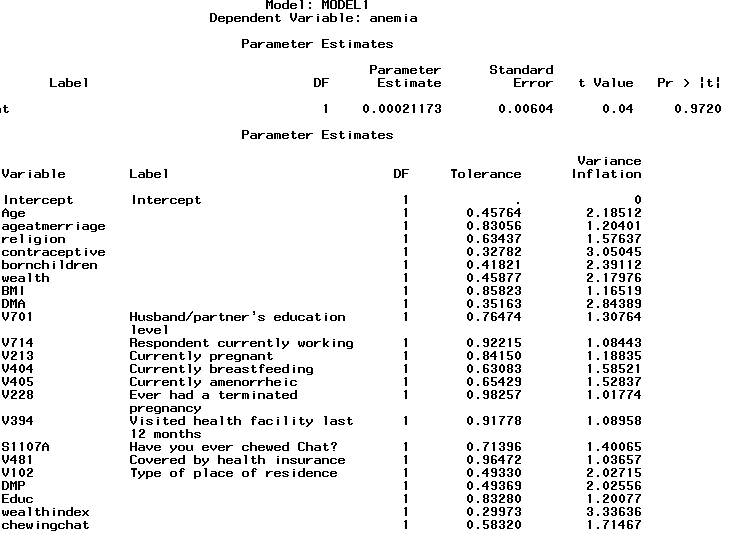


Where DMA=individual level women’s decision-making autonomy, DMP community-level women’s decision making autonomy, Educ=community-level women’s education,
